# Bullous Pemphigoid Associated with Multiple System Atrophy: Case Series

**DOI:** 10.1002/mdc3.13160

**Published:** 2021-02-25

**Authors:** Andrew Snedden, Jennifer Sharif, John Newsham, Christopher Kobylecki

**Affiliations:** ^1^ Department of Neurology Manchester Centre for Clinical Neurosciences, Salford Royal NHS Foundation Trust Salford United Kingdom; ^2^ Centre for Musculoskeletal Research University of Manchester Manchester United Kingdom; ^3^ Department of Dermatology Salford Royal NHS Foundation Trust Salford United Kingdom; ^4^ Manchester Academic Health Sciences Centre University of Manchester Manchester United Kingdom

**Keywords:** multiple system atrophy, bullous pemphigoid, α‐synuclein, skin

## Abstract

**Background:**

Bullous pemphigoid (BP) is an autoimmune blistering dermatosis associated with a number of neurological conditions, including idiopathic Parkinson's disease (IPD). Only 1 case of BP in a patient with multiple system atrophy (MSA) has been reported.

**Cases:**

We report 3 cases of men with probable MSA who developed bullous pemphigoid at a latency of 4–6 years from MSA symptom onset.

**Conclusions:**

Skin α‐synuclein deposition in neurodegenerative conditions such as IPD and MSA may be a potential substrate for the exposure of BP‐related antigens. Alternatively, central neurodegeneration may expose antigens as a substrate for cross‐reactivity and BP pathogenesis. Our report suggests an association between BP and MSA, in addition to the previously documented association with IPD.

The association between neurological disorders and bullous pemphigoid (BP) has been described in the literature. Here, we describe a case series of patients seen with both multiple system atrophy (MSA) and BP in our regional MSA service, suggesting a novel association between these conditions. In this report the clinical characteristics and investigation results of the 3 patients are described, the literature linking neurodegeneration and BP reviewed, and we suggest possible hypotheses for the association between α‐synucleinopathies and BP.

## Case Series

### Case 1

A 56‐year‐old male presented with a 2‐year history of impaired right upper limb dexterity, unsteady gait, and early falls. Over the same time period he had developed urinary urge incontinence associated with erectile failure. He had a 3‐year history of dream enactment suggestive of REM sleep behavior disorder (RBD) together with snoring and early dysphagia. He had type 2 diabetes mellitus treated with metformin and had received sitagliptin and more recently, alogliptin over the previous 4 years.

On examination he had hypomimia and gaze‐evoked nystagmus. His voice was high pitched, dysarthric, and hypophonic. He had right‐sided postural tremor but no rest tremor. There was moderate rigidity on the right side more so than on the left and bradykinesia in all 4 limbs. Lower limb reflexes were brisk and the right plantar response was extensor. Blood pressure was 140/80 lying, and 116/68 standing after 3 min.

He showed a poor levodopa response and was diagnosed with probable MSA of parkinsonian type (MSA‐P) according to consensus criteria.^1^ Sleep studies confirmed moderate obstructive sleep apnea.

**FIG. 1:**
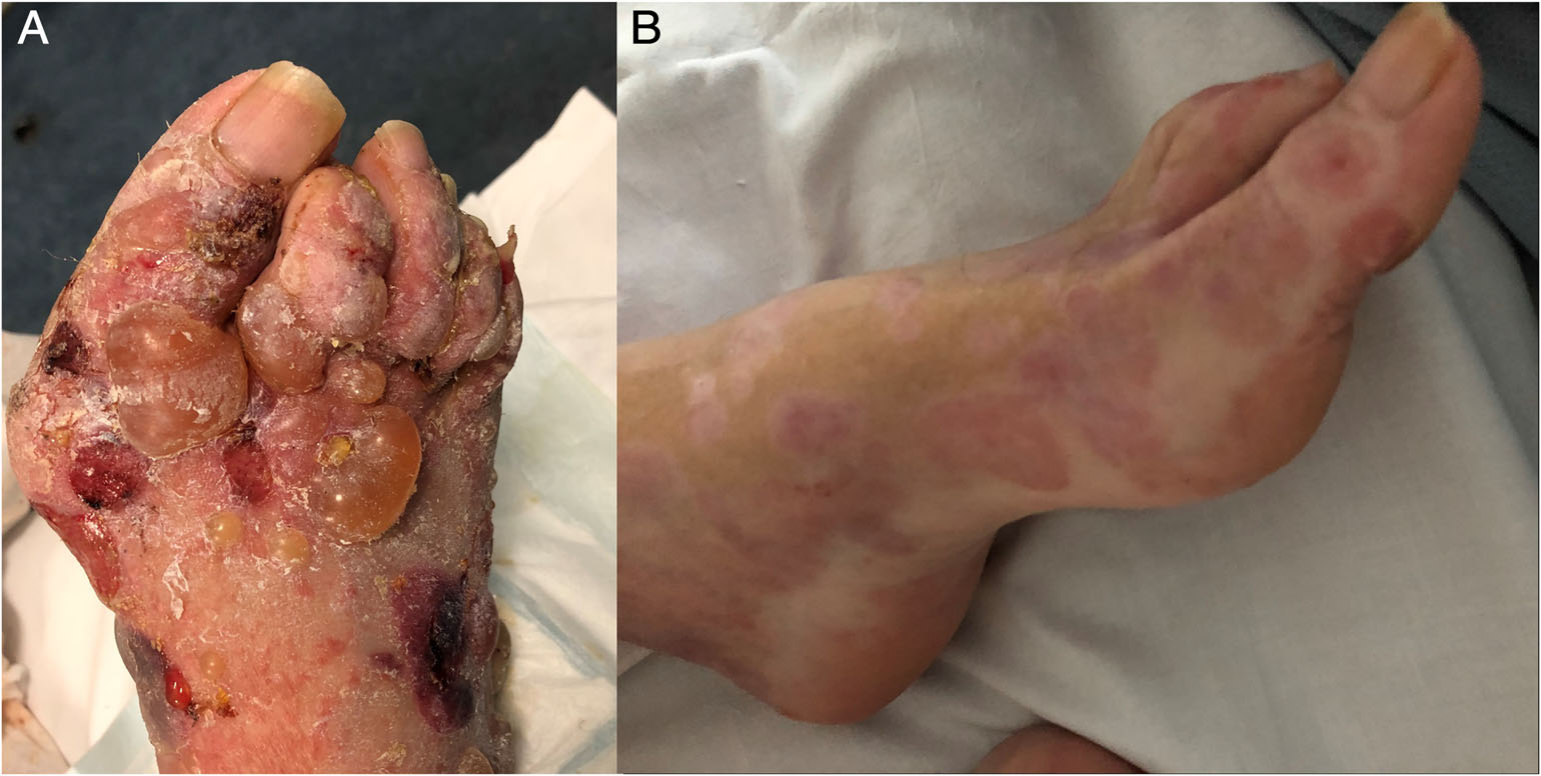
Images from case 1 at presentation (**A**), illustrating tense blisters on the right foot; blisters were widespread and bilateral on the feet at presentation. Image (**B**) shows a close of up of the left foot illustrating marked clinical improvement following immunosuppressive treatment.

Six years following symptom onset, he developed a widespread pruritic blistering rash. By this stage, he was using a wheelchair and unable to mobilize independently. On examination, he had erosions on the face and scalp; intact blisters on an erythematous base were seen on the limbs (Fig. 1A). Some of the blisters appeared hemorrhagic, and there was evidence of secondary excoriation. Nikolsky's sign was negative throughout the skin. Peripheral eosinophil count was markedly raised at 6.4 × 10^9^/L (normal range 0–0.4).

Skin biopsy showed a sub‐epidermal blister with immunofluorescence revealing evidence of linear IgG and C3 deposition at the base and roof of the blister. Indirect immunofluorescence testing on serum showed anti‐basement membrane staining. Clinical appearances and investigation results were consistent with a diagnosis of bullous pemphigoid. Control of his skin was achieved with super potent topical steroids and oral doxycycline 100 mg b.d., with significant improvement in blistering at follow‐up (Fig. 1B).

### Case 2

A 61‐year‐old‐male presented with a 12‐month history of unsteadiness of gait, erectile dysfunction, and urinary incontinence. He had a history of dream enactment suggestive of RBD. His past medical history included ankylosing spondylitis. On examination he had cerebellar dysarthria, mild limb ataxia, and moderate gait ataxia, with brisk lower limb reflexes. There was no initial postural drop in blood pressure, but this was observed within a year of diagnosis (Table 1). His mobility deteriorated rapidly, requiring a wheelchair 2 years after initial presentation.

**TABLE 1:** Details of patients with MSA and BP

	Case 1	Case 2	Case 3
Age at diagnosis (yr)	56	61	64
Symptom duration at diagnosis (yr)	2	1	1.5
Diagnosis at last clinic review	Probable MSA‐P	Probable MSA‐C	Probable MSA‐P
Autonomic involvement (most marked blood pressure changes during disease course)	Drop in SBP >30 mm Hg Drop in DBP >15 mm Hg Early urinary incontinence and erectile failure	Drop in SBP >20 mm Hg Drop in DBP >15 mm Hg Early urinary incontinence and erectile failure	Drop in SBP >30 mm Hg Drop in DBP >15 mm Hg Progressive urinary incontinence
Levodopa response	None to 800 mg/day	N/A	Transient response to 400 mg/day
MSA symptom duration at BP onset (yr)	6	4	5
Comorbidities	Type II diabetes mellitus	Ankylosing spondylitis	None
Family history of movement disorder	No	No	No
Medication at BP onset (duration)	Amantadine 100 mg daily (4 yr) Alogliptin (6 mo) Previous treatment with sitagliptin for 3.5 yr	Amantadine 100 mg daily (1 yr)	Co‐beneldopa 125 mg tds (5 yr) Amantadine 100 mg bd (5 yr)
Imaging findings	Cerebellar atrophy	Ponto‐cerebellar atrophy Hot cross bun sign Middle cerebellar peduncle T2 hyperintensity	Ponto‐cerebellar atrophy [^18^F]‐FDG PET hypometabolism in cerebellum and putamen

Antiparkinsonian medications and those with potential reported associations with BP are reported.

BP, bullous pemphigoid; MSA, multiple system atrophy; DBP, diastolic blood pressure; FDG, fluorodeoxyglucose; SBP, systolic blood pressure.

Around 4 years following symptom onset, he developed blistering of his body and lower limbs, diagnosed as bullous pemphigoid. This required systemic immunosuppression with prednisolone and azathioprine. He experienced a further flare of his skin problems 6 months later. One year after the onset of BP his skin condition was improved. He died 8 years following MSA symptom onset at the age of 68.

### Case 3

A 64‐year‐old male presented with 18 months history of impaired balance, stiffness, and reduced dexterity in the left upper limb. He reported urinary urgency preceding motor symptoms, and lightheadedness. He had several years’ history of likely RBD. His background medical history was unremarkable.

On examination, he had facial hypomimia and dysarthria, with gaze evoked nystagmus on horizontal gaze. Axial rigidity was present, with left greater than right moderate limb bradykinesia and rigidity. There was spasticity in both lower limbs with globally brisk reflexes and bilaterally extensor plantar responses. He had mild lower limb and gait ataxia. Blood pressure was 124/70 lying and 110/60 standing after 3 min.

Around 5 years following symptom onset, he developed itchy blisters on his inner thighs and abdomen. At this point, he was unable to stand and required assistance with all care. He was seen in his local dermatology service and diagnosed with BP. Control of skin disease was achieved with potent topical steroids. He died around 7 years from symptom onset at the age of 70.

## Discussion

BP is an autoimmune bullous dermatosis, which typically develops in elderly patients with a mean age of onset of 80 years. Sub‐epidermal blisters occur because of autoantibodies directed against hemidesmosomes, which bind the epidermis to the dermis. Patients develop antibodies to BP antigen 1 (BP230) or BP antigen 2 (BP180) against hemidesmosomal component macromolecules. There is increasing evidence for a link between BP and idiopathic Parkinson's disease (IPD), as well as neurological diseases, including other neurodegenerative conditions such as Alzheimer's disease.^2^ A recent systematic review of BP and neurological disease showed that BP patients had a higher risk of IPD (odds ratio [OR]: 3.06; 95% confidence interval [CI]: 1.97–4.77).^3^ A subsequent Korean study confirmed this association (OR: 3.45; 95% CI: 1.49–7.98).^4^ The standardized mortality ratio was found to be higher in BP patients when compared to the general population but there was no significant increase in mortality in BP patients under 70 years of age or with a neurological disease co‐morbidity.^4^

Despite this evidence of an association between IPD and BP, there are very few reports in the literature of links with other α‐synucleinopathies. Okazaki and colleagues reported a 63‐year‐old male with clinically diagnosed MSA who developed BP after 3 years disease duration, although the neurological features were not fully described.^5^ The only other reported description in MSA of which we are aware is a case of cicatricial ocular pemphigoid, in which other cutaneous manifestations were absent.^6^

One hypothesis for the association between BP and α‐synucleinopathies is that central neurodegeneration exposes the neuronal isoform of BP‐related antigens to the immune cells, leading to the production of autoantibodies, which cross react with the epithelial isoform.^7^ The association with other neurodegenerative disorders would support this hypothesis. BP180 protein is expressed in a variety of central nervous system (CNS) sites, and in particular, BP180 antibodies bind to tyrosine hydroxylase‐positive substantia nigra cells.^7^ No differences in titers of BP180 or BP230 antibodies were identified in patients with IPD and BP compared to those with BP alone, although those with IPD had an older age of BP onset.^8^ Deposition of phosphorylated (p‐) α‐synuclein protein has been observed in the skin of patients with both IPD and MSA. Whereas patients with IPD have significant deposition in autonomic nerve fibers, p‐α‐synuclein in MSA is located mainly in non‐synaptic subepidermal nerve fibers.^9^, ^10^ In addition, a proximal–distal gradient of p‐α‐synuclein skin deposition is seen in IPD, whereas the converse is seen in MSA.^10^ It is, therefore, plausible that cross‐reactivity to BP‐related antigens may also result from degenerative changes in skin in MSA and IPD, although there is no clear association between BP and peripheral nerve disorders.

Several drugs are known to be associated with the development of BP, although none of the antiparkinsonian agents are strongly linked to this complication.^11^ Dipeptidyl peptidase‐4 inhibitors (DPP4I) used in diabetes management have been associated with BP. However, alogliptin, which was taken by case 1 at the time of BP presentation, has not been definitively linked to this complication.^11^ In addition, marked eosinophilia has been found to be more common in idiopathic BP compared to drug‐induced BP.^12^ We cannot exclude the possibility that prior or current DPP4I use may have unmasked a tendency to develop BP as a result of MSA. However, BP in case 1 was managed without requiring withdrawal of alogliptin, suggesting that this was not a pure drug‐induced syndrome.

**FIG. 2:**
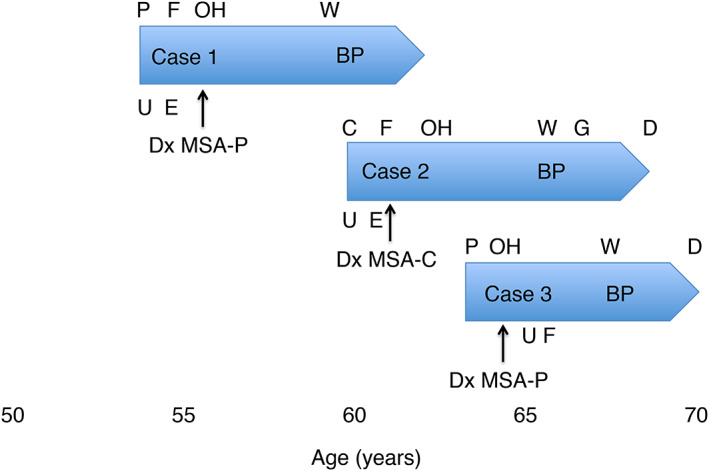
Timeline of clinical features and disease milestones in relation to bullous pemphigoid (BP) onset in all 3 cases. MSA‐P, multiple system atrophy, parkinsonian type; MSA‐C, multiple system atrophy, cerebellar type; dx, diagnosis; P, parkinsonian symptoms; C, cerebellar symptoms; U, urinary incontinence; E, erectile failure; F, falls; OH, orthostatic hypotension; W, wheelchair dependence; BP, bullous pemphigoid onset; G, gastrostomy; D, death.

A limitation of our work is the lack of pathological validation of MSA. However, all met diagnostic criteria for probable MSA^1^ and were diagnosed by the same experienced movement disorder specialist. All patients in addition had a minimum of 6 years close clinical follow‐up (Fig. 2), and had supportive imaging evidence of MSA (Table 1).^1^ A further potential limitation is that we only had access to histopathological confirmation of BP in 1 patient; however, the other 2 cases had been diagnosed by dermatology specialists. Onset of BP occurred between 4 and 6 years following MSA symptom onset, consistent with previous reports of BP following neurological disease in the majority of cases with a median latency of 5.5 years.^13^ In all 3 cases, MSA was advanced to the point of wheelchair dependence at the time of BP onset, suggesting an association with more advanced neurodegeneration. The age of onset of BP in these patients was younger than typically encountered. This case series expands on a previously reported case of BP in MSA, expanding the spectrum of BP‐associated neurodegenerative disease to MSA as well as IPD. This report suggests that further observations in larger cohorts could clarify the relationship between MSA and BP.

## Author Roles

(1) Research Project: A. Conception, B. Organization, C. Execution. (2) Manuscript Preparation: A. Writing of the First Draft, B. Review and Critique.

A.S.: 1B, 1C, 2A, 2B

J.S.: 1C, 2B

J.N.: 1C, 2B

C.K.: 1A, 1B, 1C, 2B

## Disclosures

### Ethical Compliance Statement

The authors confirm that the approval of an institutional review board was not required for this work. Verbal informed consent has been obtained to publish these cases. We confirm that we have read the Journal's position on issues involved in ethical publication and affirm that this work is consistent with those guidelines.

### Funding Sources and Conflict of Interest

The authors report no specific funding for this study, and no conflict of interest.

### Financial Disclosures for the Previous 12 Months

A.S., J.S., and J.N. report no disclosures. C.K. has received grants from Parkinson's United Kingdom and the Michael J. Fox Foundation, speaker fees from Britannia and Bial Pharma, and support to attend international meetings from Abbvie.
